# Telogen Effluvium and Anesthesia Considerations: A Case Report

**DOI:** 10.7759/cureus.88366

**Published:** 2025-07-20

**Authors:** Elizabeth Johnson-Gray, Samantha A Currier, Hanen Fernandez, Matt Brown, Matthias Franzen

**Affiliations:** 1 Anesthesiology, OhioHealth Doctors Hospital, Columbus, USA; 2 Osteopathic Medicine, Kansas City University, Kansas City, USA; 3 Internal Medicine, OhioHealth Doctors Hospital, columbus, USA

**Keywords:** acute telogen effluvium, general anesthesiology, non-scarring hair loss, telogen effluvium, telogen effluvium (t.e.) hair loss

## Abstract

Telogen effluvium is a form of non-scarring hair loss characterized by premature shedding of hair. Pathophysiologically, telogen effluvium occurs when a significant number of hair follicles prematurely enter the telogen phase, resulting in excessive shedding. The exact mechanism is unclear, but it is believed to involve alterations in the hair follicle’s growth cycle, possibly mediated by illness, infection, major surgery, low protein intake, or hormonal changes. Some medications can also be linked to this disease, such as beta blockers, carbamazepine, and vitamin A. When affected hairs are in the resting phase, noticeable hair shedding occurs. Our patient was a 61-year-old female with a history of telogen effluvium, and exacerbations occurred after surgery. She presented for a reduction and internal fixation of her left tibia and left lateral meniscus repair after a fall. She was very concerned about the possibility of undergoing surgery, as recent exacerbations had caused significant hair loss over several months.

There are no guidelines on anesthesia considerations for patients with this rare disorder. This case report hopes to shed light on techniques to reduce exacerbations in these patients.

## Introduction

Telogen effluvium is a type of hair loss that occurs when a large number of hair follicles prematurely enter the telogen (resting) phase of the hair growth cycle. This condition often results in noticeable hair shedding and thinning. Hair growth occurs in three cycles: anagen (the growth phase), catagen (the transitional phase), and telogen (the resting phase) [1,2]. When there is a disruption in this cycle, and a significant number of hair follicles (up to 70%) shift into the telogen phase, there is rapid and excessive shedding. Some reports state that those with this condition can lose 300 strands of hair per day [3,4].

This disorder can affect individuals of any age, gender, or racial background. While the exact prevalence is unknown, women tend to be more affected. There are various causes of telogen effluvium. Drugs, such as beta blockers, angiotensin-converting enzyme (ACE) inhibitors, anticonvulsants, antidepressants, and anticoagulants like heparin, have been linked to acute exacerbations. Other causes include pregnancy, states of increased cortisol, emotional stress, medical conditions like HIV, influenza, and syphilis, and physiological stress such as surgery [1,5]. Patients often present to their providers complaining of excessive hair loss, tenderness, itching, and burning on their scalp. Diagnostic tests include the modified wash test and scalp biopsy.

Treatment includes medications such as minoxidil, finasteride, avoidance of known triggers, and novel treatments such as caffeine, niacinamide, panthenol, dimethicone, and an acrylate polymer (CNPDA) [3,4]. Hair loss can be devastating and can dent a person's self-esteem and negatively affect their overall quality of life [3,4]. While patients can take control of this condition to the best of their ability, physical stressors like surgery can send a patient into an acute crisis. While anesthesia itself does not directly cause telogen effluvium, managing pain, hydration, and anxiety before, during, and after surgery can help reduce overall stress levels and reduce the chances of acute exacerbation. Currently, there are no guidelines or reports on techniques to minimize surgical stress in these patients. This case report will discuss a patient diagnosed with telogen effluvium and propose potential suggestions to reduce the risks of exacerbation.

## Case presentation

A 61-year-old Caucasian woman weighing 63.5 kg, with a body mass index (BMI) of 26.4, calculated as weight in kilograms divided by height in meters squared, has a medical history of telogen effluvium, which was previously exacerbated after an appendectomy five years ago, postoperative nausea and vomiting (PONV), asthma, and an American Society of Anesthesiologists (ASA) physical status classification of II. She presented for a reduction and internal fixation of her left tibia and left lateral meniscus repair after a fall. Upon presentation, the patient expressed concern about her upcoming surgery, noting that she experienced significant postoperative hair loss following her previous procedure. After reviewing the known triggers she mentioned, such as prolonged UV light exposure, anxiety, and pain, the team came up with a pretreatment and intraoperative plan to reduce the risk of exacerbations.

Preoperatively

Preoperatively, the team created a treatment plan that took into consideration the patient's history of PONV and asthma. The patient received aprepitant, a 1-L bolus of lactated Ringer’s solution, and nebulized albuterol. To help mitigate the risk of acute telogen effluvium exacerbation related to pain and anxiety, she was pretreated with 2 mg of midazolam and administered 20 mL of 0.5% ropivacaine for a femoral nerve block.

Intraoperatively

On induction, the patient received 200 mg of propofol, 30 mcg of sufentanil, and 100 mg of succinylcholine. Beta blockers were avoided, as they have been associated with exacerbations of telogen effluvium [1]. The patient was intubated with a 7.0 mm cuffed oral endotracheal tube. Initial end-tidal CO₂ was 32 mmHg, and she was maintained on volume control ventilation. General anesthesia was maintained with a total intravenous anesthetic (TIVA) (150 mcg/kg of propofol) to reduce her PONV, and bispectral index monitoring (BIS) was utilized to determine the depth of anesthesia. She received 8 mg of dexamethasone at the start of the case for PONV prophylaxis and 8 mg of ondansetron 30 minutes before emergence. A Bair Hugger was placed to ensure normothermia, since this can also cause an exacerbation. The temperature range was kept strictly at 36.5-37.5 degrees Celsius and monitored with a nasopharyngeal probe. She was positioned supine, with her hair wrapped in a silk scarf to prevent tearing and damage.

Since UV light is a possible contributor to telogen effluvium exacerbations, the operating room (OR) lights were dimmed, and an additional surgical drape was used to limit exposure to the patient’s face, arms, and torso. A Foley was placed to monitor urine output. She was given a total of 2.3 L of lactated Ringer's over three hours, with the urine output being 500 mL. She was normotensive throughout the procedure; therefore, no vasopressors were given.

During the procedure, she received an additional 20 mcg of sufentanil, for a total of 50 mcg, along with 30 mg of ketorolac. She tolerated the procedure well, with minimal blood loss estimated at 100 mL, and was extubated without complications. In the postanesthesia care unit (PACU), she denied any nausea or vomiting and was given a 1-g dose of intravenous (IV) acetaminophen and 1 g of Robaxin for pain control. She was admitted overnight for observation and discharged home the next day. On her follow-up clinic appointment two weeks later, she stated her pain was well controlled, and so far, she had no hair loss.

Follow-up phone calls by the anesthesia team were conducted at one month, three months, and six months after surgery. One month after surgery, she reported no hair loss. On the three-month check-in, she reported mild hair loss, stating she experienced some hair loss during the day at work, less than could fill her hand, per patient report. On her six-month follow-up, she denied any further hair loss, and the team informed the patient of the techniques used to avoid an exacerbation. She was appreciative of the team's efforts and stated that she would keep a list of the necessary measures, hoping that, should she require surgery again, her anesthesia providers could utilize some of the techniques described above.

## Discussion

Telogen effluvium is a condition of temporary hair loss that occurs when a large number of hair follicles enter the telogen (resting) phase of the hair growth cycle prematurely. The hair growth cycle has three stages: anagen (growth), catagen (transitional), and telogen (resting) [1]. Normally, about 85%-90% of hair follicles are in the anagen phase, where hair is actively growing, while only around 10%-15% are in the telogen phase. In telogen effluvium, this balance is disrupted and a higher percentage of hair follicles shift into the telogen phase, leading to noticeable shedding [4,5].

In telogen effluvium, a stressor - physical, emotional, or internal - causes a large number of hair follicles to prematurely transition to the telogen phase, leading to excessive shedding. Hair may come out in clumps during washing or brushing [6]. It typically manifests within two to three months after the triggering event, and hair loss may last for several months before regrowth begins [2,7]. It is important to be able to distinguish telogen effluvium from androgenetic alopecia to initiate the correct therapies and precautions [8]. 

The most important aspect of managing telogen effluvium is identifying and managing the triggers. One of the most common triggers for telogen effluvium is stress, especially emotional stress due to illness, surgery, or significant life changes. Chronic pain, such as post-surgical pain or the discomfort from an underlying condition, can exacerbate emotional distress. The stress associated with persistent pain can contribute to hair loss by disrupting the normal hair growth cycle and potentially triggering or worsening telogen effluvium.

While various treatment options exist such as minoxidil and finasteride, it is recommended to avoid triggers for this condition [1,3]. However, this can be challenging when patients present for elective or emergent surgery. This report hopes to highlight strategies united by the anesthesia team to decrease the risk of exacerbation. Baseline documentation on existing hair status, nutritional assessment and psychological support can be helpful starters to mediate exacerbation.

Focusing on pain control with both regional anesthesia and multimodal medication management can help reduce physical stress that can lead to an exacerbation. Continuation of a patient's home steroid medication and supplementation intra operatively can help reduce inflammation. Lastly, psychological support, such as acknowledgment of concerns to migrate anxiety may be beneficial in treating patients with a history of post-anesthetic telogen effluvium. 

## Conclusions

There are currently no guidelines on anesthesia considerations for patients with this rare disorder. Focusing on pain control, steroid use, and overall reduced emotional and physical stress may be beneficial in treating patients with a history of post-anesthetic telogen effluvium. This case report hopes to shed light on techniques to reduce exacerbations in these patients, but more research needs to be conducted to determine definitive treatment for this concern.
